# Revisional Laparoscopic Sleeve Gastrectomy in failed gastric banding and effects of exercise and frequent sweet-eating on its outcome

**DOI:** 10.12669/pjms.333.12874

**Published:** 2017

**Authors:** Hamed A. AlWadaani, Abdul Qadeer

**Affiliations:** 1 Dr. Hamed A. AlWadaani, PhD. Department of Surgery, King Faisal University College of Medicine, Al-Ahsa 31982, Kingdom of Saudi Arabia; 2 Dr. Abdul Qadeer, FCPS. Department of Surgery, King Faisal University College of Medicine, Al-Ahsa 31982, Kingdom of Saudi Arabia

**Keywords:** Bariatric surgery, BMI, Gastric banding, LAGB, Obesity, Sleeve gastrectomy

## Abstract

**Objective:**

To find out effectiveness of revisional laparoscopic sleeve gastrectomy (RLSG) in the patients who had laparoscopic adjustable gastric banding (LAGB) and failed to reduce or regained the weight and effectiveness of sweet abstaining and exercise on postoperative weight loss.

**Methods:**

This retrospective observational study was conducted at AlMoosa Hospital, Al-Ahsa, Kingdom of Saudi Arabia from December 2011 to November 2016. The patients who failed to reduce, regained the weight or had complications after LAGB, were performed RLSG. They were followed-up at three, six, twelve and twenty-four months intervals. Their weight, percent excess weight loss (%EWL) and body mass index (BMI) at pre-RLSG were compared with post-RLSG. The data was recorded in SPSS 22 and analyzed.

**Results:**

Thirty-six patients with male/female ratio of 1:5 underwent RLSG. Twelve (33.3%) were frequent sweet-eaters and twenty-four (66.7%) were not. Fourteen (38.88%) did not have exercise, while twenty-two (61.11%) had daily regular exercise. Their mean pre-RLSG weight, percent excess weight loss (%EWL)and BMI were compared with post-RLSG at the period of three, six, twelve and twenty-four months. Their mean weight reduced from 111.69 kilograms to 96.94, 87.25, 79.56 and 76.11 kilograms respectively. Their mean of the percent excess weight loss (%EWL) reduced to 22.08, 45.75, 59.64 and 66.42 kilograms respectively. Their mean pre-RLSG BMI was 43.50 kg.m^-2^, which reduced to the mean of 37.79, 34.02, 30.97 and 29.70 respectively. There were no post-operative complications in thirty (83.3%), mild like wound infection and seroma in four (11.1%) and bleeding in two (5.6%) patients. None of the patients had leakage. The patients who kept themselves abstained from sweet consumption and performed regular postoperative exercise had better results. They also had considerable reduction in appetite after RLSG.

**Conclusion:**

RLSG is an effective procedure after failed LAGB in terms of weight loss having minimal rate of complications. Moreover, abstaining from sweet consumption and continuing exercise postoperatively has better results.

## INTRODUCTION

Obesity has emerged as a leading problem of the modern world. It does not impose the cosmetic effects only but the co-morbidities like diabetes mellitus, hypertension, obstructive sleep apnea, dyslipidemia, gastroesophageal reflux disease, degenerative joint disease; depression and cancers are the main issues too.^1^ The patients are usually advised to reduce the weight by changing their life styles including diet and exercise but these modalities have usually been found ineffective in reducing the weight.^2^ Pharmacologic treatment of obesity is associated with variety of complications like peripheral neuropathy, myocardial infarction, stroke and others.^3^ Surgery has emerged as an alternative. There are many surgical procedures performed as a treatment of obesity; none is without complications. These are restrictive, malabsorptive or mixed procedures. Malabsorptive procedures are more effective in terms of weight loss and treating co-morbidities and are reserved for severe cases. The choice of the procedure depends upon the simplicity of the procedure, less complications, body mass index (BMI) and surgeon’s experience. Restrictive procedures as vertical gastroplasty and adjustable gastric banding (AGB) are the most frequently performed operations worldwide in those patients whose BMI is <50, while sleeve gastrectomy (SG) has proved as the best procedure in super obese patients.^4^ Most of these procedures are performed laparoscopically now a days. Laparoscopic adjustable gastric banding (LAGB), though it is simple, but associated with complications like dysphagia, reflux due to band migration, erosion of the stomach and failure to reduce the weight significantly. Hence, revision procedure is required. Laparoscopic sleeve gastrectomy (LSG) is considered as better option for patients who have failed LAGB.^5^ The aim of this study was to find out the feasibility, safety and long-term efficacy of revisional laparoscopic sleeve gastrectomy (RLSG) when LAGB leads to complications and fails to reduce the weight; and effects of exercise and excessive sweet consumption on weight loss.

## METHODS

This retrospective observational study was conducted at AlMoosa hospital, Al-Ahsa, Kingdom of Saudi Arabia on patients who underwent RLSG from December 2011 to November 2016 and have completed their postoperative follow-up of at least two years until November 2016. AlMoosa hospital is a tertiary care hospital having 220 beds. It is a center of excellence for bariatric surgery with single team headed by the corresponding author.

Thirty-six patients, included in this study, whose LAGB was performed somewhere else and reported to this hospital were revised with LSG. The surgery was performed in one or two steps. In patients with two-steps procedure, the band was removed as first step and RLSG was performed as second step after the interval of three to six months. Their pre-LAGB data was not completely available. During post-LAGB period, the patients either regained the weight or failed to reduce the weight considerably and some of the patients developed complications due to band. The time interval between primary LAGB and RLSG ranged between four months to eight years. The complications of band included feeling of fullness of stomach associated with epigastric discomfort/pain, reflux, dysphagia and band migration/erosion. All the patients were investigated by upper GI endoscopy, gastrografin contrast studies of GIT and CT scan of abdomen along with routine blood and biochemistry tests. Deep vein thrombosis prophylactic measures were taken in all patients by giving them low-molecular weight heparin enoxaparin (Clexane) 40-60 mg subcutaneously once a day. It was started one day before surgery and continued up to two weeks postoperatively. Intraoperative intermittent pneumatic devices were applied and continued up to twenty-four hours post-operatively. Early mobilization was encouraged in all patients. Revisional laparoscopic sleeve gastrectomy (RLSG) was performed in a standard way through four ports. During post-RLSG follow-up at three, six, twelve and twenty four months, the patients were asked about their food habits, change in appetite, exercise, any complications and their weight was recorded. All the data were recorded in SPSS version 22 and analyzed.

The ‘ideal BMI’ to be achieved after RLSG was set at 22.5 kg.m^-2^, though there was still room to reduce further weight to reach the lower level of the normal BMI. Hence, the percent excess weight loss (%EWL) was calculated at three, six, twelve and twenty-four months intervals by the following formula:^6^


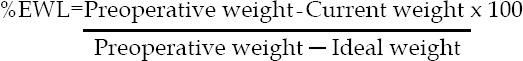


## RESULTS

Out of total thirty-six patients, six (16.7%) were males and thirty (83.3%) were females. Age of the patients ranged between 18 to 52 years (mean = 30.67 and median = 29 ± 8.77). Eight patients (22.2%) were obese (BMI 36.3-39.8 kg.m^-2^). Sixteen (44.4%) were morbid obese (BMI 40.2-44.8 kg.m^-2^). Eight (22.2%) were super-obese (BMI 45.1-49.2 kg.m^-2^). Four (11.1%) were super-super-obese (BMI 51.3-56.6 kg.m^-2^). Their mean and median BMI were 43.5 and 42.1 respectively. After primary LAGB, twenty-eight patients had regained the weight over variable period of time, six failed to reduce the weight significantly, one had migration of the band and one had gastric erosion. Twelve (33.3%) were frequent sweet-eaters and twenty-four (66.7%) were not. Fourteen patients (38.88%) did not have post-operative exercise, while twenty-two (61.11%) had regular daily exercise.

The interval between primary LAGB and RLSG ranged between four months to eight years (Mean = 36.83 months). Surgery was performed in single step in twenty-eight (77.8%) and two steps in eight (22.2%) patients. There were no post-operative complications in thirty (83.3%), mild like wound infection and seroma in four (11.1%) and bleeding in two (5.6%) patients. None of the patients had leakage. The mean pre-RLSG weight was 111.69 kilograms, which reduced to 96.94, 87.25, 79.56 and 76.11 kilograms at the post-RLSG period of three, six, twelve and twenty-four months respectively (Table-I).

**Table-I:** Comparison of weight, BMI and %EWL pre-RLSG with post-RLSG at different times.

*Parameter*	*Pre-RLSG*	*Post-RLSG*			

		*At 3-months*	*At 6-months*	*At 12-months*	*At 24-months*
***Weight loss (kg)***					
•Mean	111.69	96.94	87.25	79.56	76.11
•Median	109.50	95.50	86.50	78.00	72.00
•Std. Deviation	15.432	13.984	11.953	11.090	12.549
***BMI loss (kg.m^-2^)***					
•Mean	43.50	37.79	34.02	30.97	29.70
•Median	42.15	37.08	33.65	30.64	29.67
•Std. Deviation	5.0119	4.80060	4.17127	4.22942	4.62666
***%EWL (kg)***					
•Mean	--	28.08	45.75	59.64	66.42
•Median	--	25.50	47.00	63.50	72.00
•Std. Deviation	--	10.932	13.770	17.258	20.325

**Fig.1:**
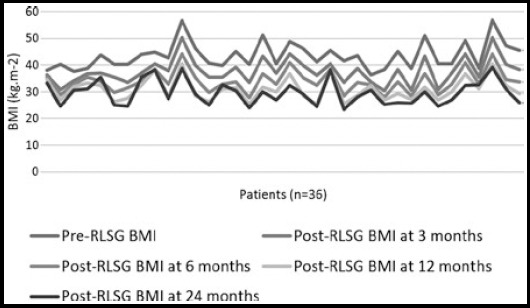
Pre- & post-RLSG BMI at different time intervals.

**Fig.2:**
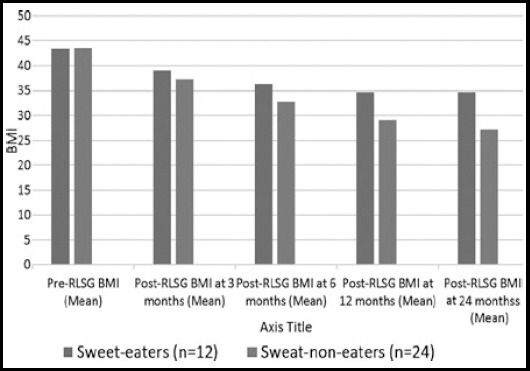
Comparison of BMI between frequent sweet-eaters & non-eaters.

**Fig.3:**
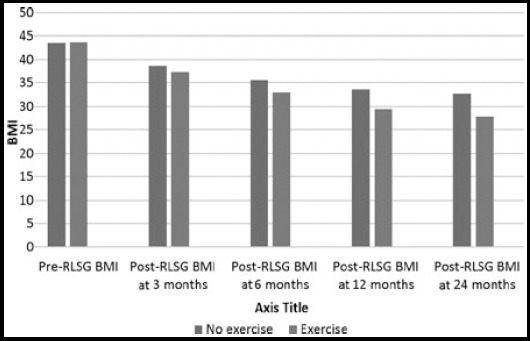
Comparison of BMI with and without exercise

The findings in the study show that post-operative weight loss depends upon the pre-operative weight. More the pre-operative weight more is the weight loss post-operatively (Fig.1). The mean of the percent excess weight loss (%EWL) was 22.08, 45.75, 59.64 and 66.42 kilograms at the period of three, six, twelve and twenty-four months respectively after the RLSG (Table-I). Mean pre-RLSG BMI of the patients was 43.50 (Range = 36.3 to 57.0) which reduced to the mean of 37.79, 34.02, 30.97 and 29.70 at the period of three, six, twelve and twenty-four months respectively after RLSG (Table-I). Seven patients (19.44%) though initially reduced the weight after RLSG, but afterwards they either failed to reduce further or started gaining weight around the period of two years. This was observed more in those patients who were frequent sweet eaters and/or not doing/stopped regular exercise (Fig.2 & 3). Twenty-six (72.22%) patients developed remarkable (25-70%) reduction in appetite after RLSG, while the remaining patients also experienced less than 25% reduction in appetite.

Thirty patients (83.33%) did not develop any post-operative complication. Four (11.11%) developed mild complications like wound infection, seroma/abscess and were dealt accordingly.

Two patients (5.55%) suffered from bleeding on first post-operative day and managed conservatively with one unit of blood transfusion and regular ultrasound of abdomen. Mean operation time was 90 (±30) minutes. Mean hospital stay of patients was three days.

## DISCUSSION

LAGB is simple, effective procedure for weight loss. It has less number of complications and easily performed within short time but it is not without complications. The loss of weight achieved by this is less than other bariatric procedures. Moreover, the rate of re-operation is more as compared to other procedures.^7^,^8^ It is usually advocated that a restrictive procedure should be converted to malabsorptive, like gastric bypass or biliopancreatic diversion with or without duodenal switch, if revision is required.^9^ But many studies have shown successful conversion of LAGB to LSG with promising results in terms of weight loss.^10^,^11^ LAGB is not found appropriate bariatric procedure in super-obese patients. It has failed to reduce weight significantly in such patients.^12^

In our study, some patients underwent revision between four to ten months of primary LAGB, because of the complications of band. Hence it shows that LAGB is not without short and/or long term complications.^13^ Gastric erosion is the worst among its complications that may lead to perforation or sometimes devastating the shape of stomach badly.

The male/female ratio in our patients was 1:5. This does not necessarily reflect the difference of rate of obesity in two sexes in Saudi Arabia but the difference of presenting themselves for surgery. It reflects more health consciousness in females about their figure. The same trend has been observed in a Nationwide Inpatient Sample database reviewed by Young MT et al in the USA, where males and females who underwent bariatric surgery were 19.3% and 80.7% respectively with M:F ratio of 1:4.^14^

The mean age in our patients was 30.67 years; which is alarming that obesity in Saudi Arabia prevails in quite younger age. This may be due to sedentary life style and consuming excessive sweets.^14^ This is evident in our study by the number of super-obese (22.22%) and super-super-obese (11.11%) patients.

Within twenty-four months of RLSG in our patients, the mean weight of 111.69 kg reduced to 76.11 kg. The mean %EWL also reduced up to 66.42 kg and mean BMI reached from 43.50 kg.m^-2^ of pre-RLSG period to 29.70 kg.m^-2^ (the mean reduction of BMI up to 13.8 kg.m^-2^). There is gradual reduction in excessive weight (EW) and BMI over the period of twenty-four months. These results are encouraging. All the above parameters prove the effectiveness of RLSG as an alternative procedure when LAGB does not suit anyway.^15^,^16^ Seven patients (19.44%) in our study failed to reduce or regained the weight (Fig.1), which is quite acceptable and comparable with other studies and can be improved with frequent follow-up and encouraging the patients to abstain themselves from sweets and continuing regular exercise.

In our study, the habit of sweet eating postoperatively has significant effect on weight loss. There is also tendency in frequent sweet-eaters to regain the weight, hence strict follow-up and advice is required (Fig.2). This matches with some studies^17^, but Moser F et al. and Hudson SM et al. in their separate studies have not found remarkable effects of preoperative sweet eating habits in terms of weight loss after sleeve gastrectomy and lap-band placement, which are contradictory to our findings.^18^,^19^

The role of postoperative exercise in obesity surgery is well established.^20^ Our study also found significant effect of exercise on weight loss. Those patients who continued their exercise on regular basis have shown remarkable weight reduction than those who did not (Fig.3).

Reduction in appetite, though is subjective, is significantly observed in our study. It is the advantage of sleeve gastrectomy that ghrelin-producing area of stomach is resected.

Post-RLSG complications are within acceptable range as compared to other studies. Studies show that the conversion to LSG from LAGB has less complications as compared to malabsorptive procedure.^21^,^22^

## CONCLUSION

RLSG is an effective procedure after failed LAGB in terms of weight loss. It has minimal rate of complications. Moreover, abstaining from sweet consumption and continuing exercise postoperatively has better results. The patients should be followed-up for longer time to keep them motivated for regular exercise and abstained from excessive sweet consumption.

### Authors’ Contribution

**HAAW:** Analyzed, edited, reviewed and finally approved the manuscript.

**AQ:** Searched and collected the data, analyzed and prepared the manuscript.
